# Socio-demographic determinants of malnutrition among primary school aged children in Enugu, Nigeria

**DOI:** 10.11604/pamj.2017.28.248.13171

**Published:** 2017-11-21

**Authors:** Obianuju Igbokwe, Gilbert Adimorah, Anthony Ikefuna, Ngozi Ibeziako, Agozie Ubesie, Christopher Ekeh, Kenechukwu Iloh

**Affiliations:** 1 Department of Paediatrics, College of Medicine, University of Nigeria, Enugu, Nigeria; 2 Department of Paediatrics, University of Nigeria Teaching Hospital, Ituku,Ozalla, Enugu, Nigeria

**Keywords:** Socio-demographic, malnutrition, school-age, children, Enugu

## Abstract

**Introduction:**

Several factors including the parental literacy, illness, socioeconomic status, poor sanitation and hygienic practices affect the physical growth of children. The aim of this study was to determine the socio-demographic determinants of malnutrition among primary school aged children in Enugu, Nigeria.

**Methods:**

A cross-sectional descriptive study involving primary school children in Enugu was carried out over a 3 month period. Subjects were selected using multistage sampling technique. Weight and height were measured using a digital scale and a wooden stadiometer, respectively. Body Mass Index (BMI), weight-for-age (WAZ), Height-for-age (HAZ) and BMI-for-age z scores were then derived using the new WHO reference standards.

**Results:**

348 children (40.4%) were recruited from 5 public schools while 512 (59.6%) were recruited from 9 private schools. The mean age of the study participants was 9.2 ± 1.8 years. 7 (0.8%) children were stunted, 26 (3.3%) wasted and 28 (3.3%) underweight. Of all the study participants, overweight and obesity were observed in 73 (8.5%) and 35 (4.1%) children, respectively. Children of lower socioeconomic class were more stunted, underweight and wasted, while overweight and obesity were more prevalent among children from the upper socioeconomic class.

**Conclusion:**

Factors such as age and sex, parental education and socioeconomic class had a significant impact on nutritional status. Overweight and obesity were more prevalent among the children from the upper socioeconomic class, attending private schools, while stunting and wasting were more in children of the lower class attending public schools.

## Introduction

Malnutrition is the most important risk factor for morbidity and mortality, contributing to more than half of child deaths worldwide. In Nigeria, school age children constitute 23% of the total population [1]. While sub-Saharan Africa is still struggling with high rates of underweight and stunting in children under five, with the proportion of stunted children being 41% [2], overnutrition is also increasing in the sub-continent. Recent reviews have reported significant increase in overweight and obesity in developing countries [3]. The nutritional status of children is a reflection of the socioeconomic status of the family and the social wellbeing of the community. Indeed, there is an established association between child nutrition and socioeconomic status. The socioeconomic status of families can usually be assessed through family income, housing conditions, parents' education, occupation, and family composition [4]. The nutritional status also portrays the efficiency of the health care system and the influence of the surrounding environment [5]. Parental literacy is a critical determinant of the well-being of children in developing countries especially maternal literacy [6]. Studies have shown that child survival, nutritional status and educational attainment are enhanced by having better-educated mothers [7-11]. In addition, a mother's level of education is an important determinant of her children's health. A high level of maternal education could lead to an increased awareness of healthy behavior, sanitation practices and a more equitable distribution of household resources in favor of the children. Educated mothers can influence the health of their children by challenging existing traditional beliefs and attitudes. In addition, these mothers also tend to exhibit a greater willingness to accept developmental initiatives and utilize modern health care [4]. Fathers' education is also an important determinant and has a positive impact on child health and nutritional status as fathers are usually the bread winners and decision makers in the most cases in the African setting. Family size has also been shown to be positively correlated with nutritional status. Pelto and his co-workers [12] examined the extent to which house hold size was related to the nutritional status of Mexican children and found that those from larger households were significantly shorter and consumed diets of poor quality. This is not surprising because the larger the size of the family, the more difficult it is for the caregiver to cater for the individual. This study sought to determine the socio-demographic determinants of malnutrition among school age children in Enugu, Nigeria.

## Methods

**Study design:** A cross sectional study of school age children in Enugu North LGA carried out between March and July 2013.

**Study area:** The study was conducted in Enugu North LGA of Enugu State Nigeria. The local government area is one of the three in Enugu metropolis. There are 139 primary schools (51 public and 88 private) with a total school population of 82,116 pupils.

**Inclusion criteria:** Children aged 6-12 years, enrolled in any of the selected registered public or private primary schools in Enugu North LGA.

**Exclusion criteria:** Children with skeletal deformities, those whose age could not be ascertained or were on medications known to affect growth such as steroids were excluded from the study.

**Sampling method:** The ratio of public to private schools in the study area was 1:1.7. Five public and nine private schools were therefore selected for the study using simple random sampling without replacement. The number of subjects selected from each school was determined using the Neymann allocation formula for stratified sampling. In each selected school, the allocated sample size was proportionately divided among the sections, and the total number of students in each section constituted the sampling frame in that section. The allotted sample was then divided according to the number of classes in each section. In each class, the participants were selected by simple random sampling using a statistical table of random numbers until the required number for the class was obtained.

**Ethical approval and consent:** Ethical approval was obtained from UNTH Health Research Ethics committee. Permission was also obtained from the Enugu State Universal Basic Education board (ESUBEB) and various head teachers of the selected schools. Written informed consent was obtained from parents of the selected pupils.

**Determination of social class:** The social class was determined using the classification proposed by Olusanya et al. [13] and stratified into five classes. Scores were awarded to each child based on the father's occupation and the mother's educational attainment. Scores of 1 and 2 were grouped under the upper class; score of 3 the middle social class, while scores of 4 and 5 were the lowest socioeconomic class.

**Data collection :** A proforma designed for the study was used to record the information obtained. The weight and height measurements were carried out according to standard procedures described by WHO. All measurements were taken with the children wearing light clothing and without shoes. Each child was weighed using a calibrated standardized digital weighing scale (OMRON BF400), with the accuracy of the scale to the nearest 0.5kg. The weight was recorded twice and the average value used in the analysis. The scale was set to zero point before each use and checked for accuracy with standard weights after every 20 measurements or whenever the scale was moved from place to place. Heights were measured with a wooden stadiometer placed on a flat surface measured to the nearest 0.1cm. Two measurements were taken and the average value was obtained. BMI was calculated using the formula:

$$BMI=\frac{\text{Weight}(\text{Kg})}{\text{Height}(M^{2})}$$

Weight-for-age, Height-for-age, and BMI-for-age were derived from the new WHO standard/ reference. Computed Z scores for BMI for age , weight for age and height for age were then used to assess underweight, wasting, stunting, overweight and obesity using the recently published WHO reference standards. Normal height was defined as height for age which is between -2 and +2 Z score while normal weight was defined as weight for age between -2 and +2 Z score. Stunting and underweight were defined as height and weight for age less than -2 Z score respectively. Wasting was defined as BMI for age less than -2 Z score while obesity was defined as BMI greater than +2 Z score. Overweight was defined as BMI for age between +1 and +2 Z score.

**Data analysis:** Data obtained was recorded and analyzed using the Statistical Package for Social Sciences (SPSS) version 18.0. Descriptive statistics which include frequency, percentages, means and standard deviations were used to summarize the variables. Logistic regression was used to determine association between categorical variables, while comparison of means between public/private schools was done using the Student's t test. All tests were 2-tailed and significant at less than 0.05.

## Results

Eight hundred and sixty (396 males and 464 females) children were included in the data analysis. Of this, 348 (40.4%) were from public schools while 512 (59.6%) were from private schools. The mean age of the study subjects was 9.2 ± 1.8 years. Majority of the pupils (78.7%) in the public school were from the lower socioeconomic class, while in the private schools, about half (54.7%) were from the upper social class as shown in Table 1.

**Table 1:** Socio-demographic characteristics of the study subjects in private and public schools

Variables	Private schools n (%)	Public schools n (%)
**Social class**		
**Upper**	280 (54.7)	26 (7.5)
**Middle**	69 (13.5)	48 (13.8)
**Lower**	163 (31.8)	274 (78.7)

**Influence of maternal education on the nutritional status of the study subjects:** As shown in **Table 2**, maternal education had significant statistical influence on the nutritional indices tested. Twenty children (4.4%) whose mothers had secondary or lower education compared to six (1.5%) whose mothers had tertiary education were wasted (p = 0.01). Conversely 54 children (13.2%) whose mother had tertiary education compared to nineteen (4.2%) whose mothers had secondary or lower form of education were overweight (p<0.001).

**Table 2:** Effect of maternal educational status on the nutritional status of the study population

Nutritional Status	Tertiary n (%)	Secondary &below n (%)	P value	OR	95% C.I
**Normal**	311 (76.0)	380 (84.3)	0.003	1.687	1.200 - 2.370
**Stunting**	2 (0.5)	5 (1.1)	0.326	0.438	0.085 - 2.272
**Wasting**	6 (1.5)	20 (4.4)	0.010	0.435	0.230 - 0.822
**Underweight**	8 (2.0)	20 (4.4)	0.047	0.430	0.187 - 0.987
**Overweight**	54 (13.2)	19 (4.2)	< 0.001	3.459	2.013 - 5.943
**Obesity**	28 (6.8)	7 (1.6)	< 0.001	4.661	2.013 - 10.792

**Influence of paternal education on nutritional status of the study subjects:** As illustrated in Table 3, wasting and underweight was more prevalent among children whose fathers had secondary education and this difference was statistically significant (p<0.05). Overweight and obesity were significantly more prevalent among children of fathers with tertiary education (p<0.001).

**Table 3:** Effect of paternal educational status on the nutritional status of the study population

Nutritional Status	Tertiary n (%)	Secondary& below n (%)	OR	p value	95% C.I
**Normal**	313 (76.2)	378 (84.2)	1.667	0.003	1.186 - 2.342
**Stunting**	2 (0.5)	5 (1.1)	0.434	0.320	0.084 - 2.250
**Wasting**	4 (1.0)	22 (4.9)	0.270	< 0.001	0.133 - 0.549
**Underweight**	7 (1.7)	21 (4.7)	0.353	0.019	0.149 - 0.840
**Overweight**	58 (14.1)	15 (3.3)	4.754	<0.001	2.649 - 8.532
**Obese**	27 (6.6)	8 (1.8)	3.876	0.001	1.740 - 8.632

**Impact of socioeconomic class on nutritional status of the study population:** Table 4 and Table 5 illustrate the relationship between socioeconomic class and the nutritional status of the study subjects. Children from the middle and lower socioeconomic classes were more underweight and wasted than children from the upper class, and this difference was statistically significant (p<0.05). Children from the upper class were more overweight and obese than children from the other socioeconomic classes. (p =0.001).

**Table 4:** Relationship between socioeconomic class and nutritional status of the study population (upper class vs. other classes)

Nutritional Status	Tertiary n (%)	Secondary& below n (%)	OR	p value	95% C.I
**Normal**	313 (76.2)	378 (84.2)	1.667	0.003	1.186 – 2.342
**Stunting**	2 (0.5)	5 (1.1)	0.434	0.320	0.084 - 2.250
**Wasting**	4 (1.0)	22 (4.9)	0.270	< 0.001	0.133 - 0.549
**Underweight**	7 (1.7)	21 (4.7)	0.353	0.019	0.149 - 0.840
**Overweight**	58 (14.1)	15 (3.3)	4.754	<0.001	2.649 - 8.532
**Obese**	27 (6.6)	8 (1.8)	3.876	0.001	1.740 - 8.632

**Table 5:** Relationship between socioeconomic class and nutritional status of the study population (lower vs. other classes)

Variable	Lower class n (%)	Other classes	OR	p value	95% C.I
**Stunting**	5 (0.8)	2 (28.6)	2.436	0.289	0.470 -12.627
**Underweight**	20 (4.6)	8 (28.6)	2.488	0.032	1.084 - 5.712
**Wasting**	22 (5.0)	4 (15.3)	0.289	0.001	0.145 - 0.574
**Overweight**	17 (3.9)	56 (76.7)	0.265	0.001	0.151 - 0.465
**Obese**	7 (1.6)	28 (80.0)	0.230	0.001	0.099 -0.532

## Discussion

In the present study, parental education was an important determinant of children's nutritional status. Children of patients with tertiary education were more likely to be obese while those whose parents had secondary or lower form of education tend to have undernutrition. The reason could be because parents without good education may not be able to afford healthy nutritious food for their children from the arrays of available food items. Parents who are better educated are more likely to be empowered financially. It's been reported that affluence is associated with overnutrition among children in developing nations like Nigeria [14]. Maternal Literacy is also an important determinant of the nutritional status of children. In the present study, a lower prevalence of undernutrition was found in children of mothers with tertiary education. This is comparable to findings by other authors who reported that higher maternal education improves the nutritional status of school children [7-11]. Alderman and Luc in Ethiopia observed that mothers with secondary education and above had a positively significant effect on the anthropometric scores of their children when compared with uneducated mothers [15]. Immink and Payongayong in Guatemala also identified maternal literacy status as an important risk factor for growth failure in children [16]. Similarly, Babar et al. in Pakistan documented higher rates of malnutrition among children of illiterate mothers compared to literate mothers [4]. This can be explained by the fact that mothers who are educated are more likely to be gainfully employed and usually have easier access to information on healthy feeding for their children. In addition, they tend to have an opinion in terms of allocation of family income in favour of the children. They are also better informed on safe and hygienic practices, water resources and seek early treatment of infections when they occur.

In the present study, the prevalence of overweight and obesity in children of mothers with tertiary education was noted to be higher than the prevalence in children whose mothers had secondary education and below. This is similar to the findings by Babar et al. in Pakistan [4]. These mothers with high purchasing power are likely to afford expensive fast foods and high-calorie diets which will increase the risk of overweight and obesity in their children. As expected, paternal education was also found to be closely associated with malnutrition. Children of fathers with secondary education and below were more wasted and underweight than children of fathers with tertiary education. This was in keeping with findings of some authors in Pakistan who documented higher rates of stunting and underweight in children whose fathers had secondary education and below than children of fathers with tertiary education [4, 17]. The reverse was the case for overweight and obesity which was found to be higher among the children of fathers with tertiary education than in the children whose fathers had secondary education and below. Socioeconomic status is also an important determinant of child nutritional status. It affects access to culturally appropriate and affordable food, thus affecting the quality of the diet. Stunting, underweight and wasting were significantly higher among children from lower socioeconomic class than those of the upper class. This was similar to the findings of several authors worldwide. In Onitsha, southeastern Nigeria, Ndukwu et al found that children from lower social class were more stunted than their upper-class counterparts [18]. Other Nigerian studies also reported higher rates of stunting and underweight in children from lower socioeconomic class than in those from the upper class [19]. In the present study, prevalence of overweight and obesity were found to be significantly higher among children from upper social class than their counterparts from the lower socioeconomic class. This is comparable to findings by several authors worldwide. However, this relationship between obesity and socioeconomic status varies across countries. For instance, the risk of obesity is more prevalent in upper socioeconomic class in Russia and China, while in the USA, obesity is more prevalent in the lower socioeconomic class [20, 21]. This has been attributed to the fact that in people of low socioeconomic status, food deprivation or fear of deprivation can lead to overeating when food is available [22].

## Conclusion

Overweight and obesity appear to be emerging as nutritional problems especially among children from upper socioeconomic class. It is therefore pertinent that caregivers and their children are educated on the benefits of optimal nutrition and balanced diet.

### What is known about this topic

- Overnutrition does occur among children is a known public health challenge in developed countries;
- It is more common among children from poor socioeconomic background in developed countries.

### What this study adds

- Confirms rising incidence of overnutrition among children from developing countries;
- Unlike developed countries, higher socioeconomic status contributes to the rising incidence.

## Competing interests

The authors declare no competing interests.
